# Temperature Stable Ion Exchange Resins as Catalysts for the Manufacturing of Vitamin Precursors by Aldol Reaction

**DOI:** 10.3390/ijms241814367

**Published:** 2023-09-21

**Authors:** Jonas Vosberg, Thomas Bouveyron, Simon Eisen-Winter, Jan Drönner, Gerhard Raabe, Pierre Vanhoorne, Sven Behnke, Matthias Eisenacher

**Affiliations:** 1 Circular Transformation Lab, TH Köln-University of Applied Sciences, 51379 Leverkusen, Germany; 2 Institute of Organic Chemistry, RWTH Aachen University, Landoltweg 1, 52074 Aachen, Germany; 3 Lanxess Deutschland GmbH, LPT-M-R&I, Kaiser-Wilhelm-Allee, 51369 Leverkusen, Germany

**Keywords:** ion exchange resin, aldol reaction, heterogeneous catalysis, vitamins, catalyst deactivation

## Abstract

This study explores the potential of robust, strongly basic type I ion exchange resins—specifically, Amberlyst^®^ A26 OH and Lewatit^®^ K 6465—as catalysts for the aldol condensation of citral and acetone, yielding pseudoionone. Emphasis is placed on their long-term stability and commendable performance in continuous operational settings. The aldol reaction, which traditionally is carried out using aqueous sodium hydroxide as the catalyst, holds the potential for enhanced sustainability and reduced waste production through the use of basic ion exchange resins in heterogeneous catalysis. Density Functional Theory (DFT) calculations are employed to investigate catalyst deactivation mechanisms. The result of these calculations indicates that the active sites of Amberlyst**^®^** A26 OH are cleaved more easily than the active sites of Lewatit**^®^** K 6465. However, the experimental data show a gradual decline in catalytic activity for both resins. Batch experiments reveal Amberlyst^®^ A26 OH’s active sites diminishing, while Lewatit^®^ K 6465 maintains relative consistency. This points to distinct deactivation processes for each catalyst. The constant count of basic sites in Lewatit^®^ K 6465 during the reaction suggests additional factors due to its unique polymer structure. This intriguing observation also highlights an exceptional temperature stability for Lewatit^®^ K 6465 compared to Amberlyst^®^ A26 OH, effectively surmounting one of the prominent challenges associated with the utilization of ion exchange resins in catalytic applications.

## 1. Introduction

The search for catalysts that promote sustainable chemistry aligns with the fundamental principles of green chemistry [1]. Within this context, the utilization of heterogeneous base catalysts in industrial applications is relatively uncommon [2]. Research work is being conducted in the field of the catalytic use of ion exchange resins, e.g., the sulfation of birch wood microcrystalline cellulose [3]. However, a promising and noteworthy category of catalysts in this domain are ion exchange resins, which showcase remarkable attributes, including high activity across various reactions. These types of catalysts exhibit the capacity to facilitate numerous chemical transformations efficiently and have already proven invaluable in water treatment processes such as water softening, complete deionization, and the purification of potable water [4,5]. Nonetheless, it is crucial to acknowledge that these catalysts can experience rapid deactivation due to the elimination or loss of the active sites [6,7,8]. This aspect presents a challenge in maintaining their long-term catalytic efficiency. The aldol reaction, which is a common reaction for the formation of carbon–carbon bonds [9,10,11], is industrially carried out using homogeneous catalysts such as sodium hydroxide or tertiary amines [12,13,14,15,16]. However, this reaction can be carried out using basic heterogeneous catalysis, which is still in development and has not been used industrially up to now [6]. Using homogeneous catalysts has some intrinsic disadvantages: The catalysts used are diluted after the reaction and must either be purified in an energy-intensive manner or disposed of as a salt. In addition, the separation of the homogeneous catalysts is energy-intensive. Furthermore, the contact time of the reactants on the catalyst can only be poorly controlled [17,18,19].

LANXESS, a leading chemical company, has developed a novel resin carrying an experimental linker that might exhibit greater thermal stability. It has primarily been utilized for anion separation according to EP 1908 521 B1; for this application, it showed superior temperature stability in comparison to other basic ion exchange resins. Therefore, its potential as a catalyst has yet to be explored [20].

Amberlyst^®^ A26 OH [21] is well known as a basic catalyst for aldolization reactions [6], hence it is used as a benchmark to evaluate Lewatit^®^ K 6465’s performance as a catalyst. Table 1 sums up the physical and chemical properties of both ion exchange resins. By analyzing the physical and chemical properties of these two resins, we can gain insights into their similarities and differences, providing valuable information for catalyst selection and process optimization.

Both catalysts are composed of a polystyrene backbone and trimethylammonium hydroxide groups as an active catalytic site, but differ in the way the ammonium group is linked to the backbone as shown in Table 2.

Two functional groups are used in anion exchangers. Depending on the type of group, a distinction is made between weakly and strongly basic ion exchangers. In the case of the weakly basic ion exchangers, the basicity depends on the Lewis basicity of the free electron pair of an amino group. In the case of the strongly basic ion exchangers, a distinction is made between type I and type II depending on their structure—both of which have a quaternary amine compound as a functional group [23].

Amberlyst^®^ A26 OH and Lewatit® K 6465 are strongly basic type I ion exchange resins.

**Type I:** R–N^+^(CH_3_)_3._

**Type II:** R–N^+^(CH_3_)_2_CH_2_CH_2_OH.

**Figure 1:**
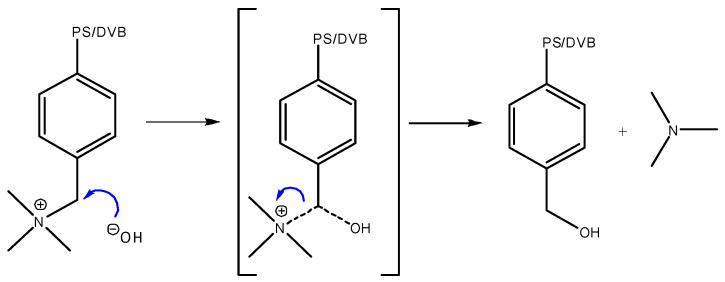
S_N_2 mechanism of base-catalyzed cleavage of anion exchange resins’ functional group. PS/DVP: Polystyrene/Divinylbenzene.

Previous studies have shown that certain resins undergo deactivation even at mild conditions. For example, Amberlyst^®^ A26 OH is known to deactivate through an S_N_2 mechanism [23,24], while Lewatit^®^ K 6465 supposedly deactivates via Hofmann elimination or in an S_N_2 reaction as well [25]. Understanding the deactivation mechanisms associated with different resins is crucial for optimizing their catalytic performance and extending their lifespan. The S_N_2 reaction is favored by the primary carbon of the functional group [26]. The S_N_2 deactivation mechanism is shown in Figure 1: a transition state is formed because of nucleophilic attack by the hydroxide ion. The positively charged amino group is a better leaving group than the hydroxide and is split off.

**Figure 2:**
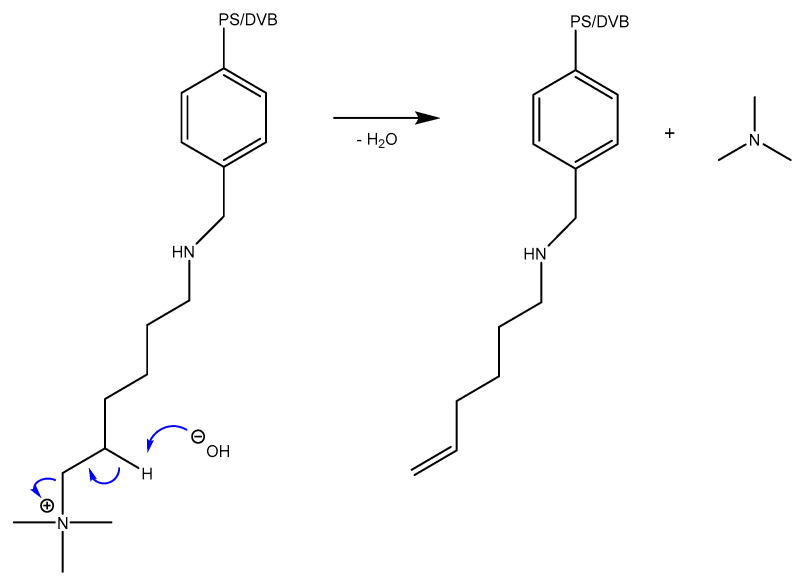
Base-catalyzed Hofmann elimination mechanism of cleavage for anion exchange resins’ functional group with a linker [27,28]. PS/DVP: Polystyrene/Divinylbenzene.

The Hofmann elimination, in the course of which the β-carbon of the linker is deprotonated by a base, results in the cleavage of an amino group molecule from the substrate, as shown in Figure 2 [9]. The remaining electron pair rearranges to the α-carbon, leading to the formation of a double bond and the generation of an alkene.

**Figure 3:**
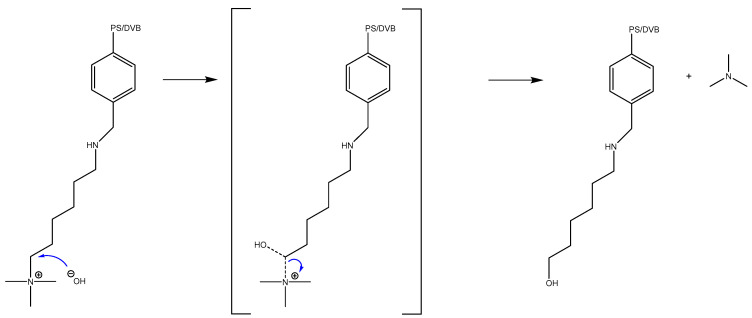
Base-catalyzed S_N_2 mechanism of cleavage for anion exchange resins’ functional group with a linker. PS/DVP: Polystyrene/Divinylbenzene.

The other possible mechanism for the deactivation of Lewatit^®^ K 6465 is the S_N_2 reaction analogous to the deactivation of Amberlyst^®^ A26 OH, as described in Figure 3.

The elimination is favored with the increasing temperature. This results in a maximum operating temperature of 60 °C for the hydroxide forms of the strongly basic ion exchangers of type I and 35 °C for the ion exchangers of type II. For the chloride form, the maximum operating temperature is 80 °C for type I and 60 °C for type II [25]. We propose that the introduction of a crosslinker alters the mechanism by which the catalyst deactivates resulting in improved thermal stability and over all durability.

**Figure 4:**
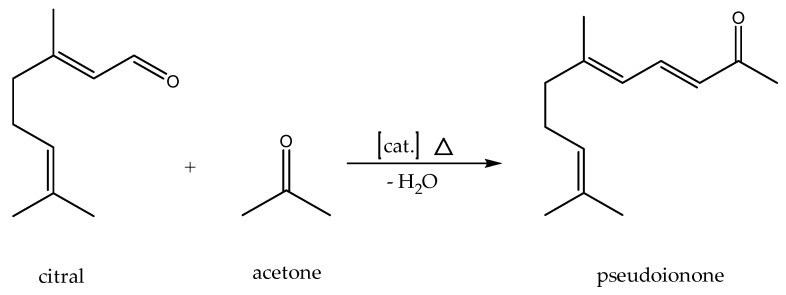
Reaction of citral and acetone to pseudoionone.

As an aldol reaction of industrial interest for investigating the catalytic activity of both ion exchange resins, we have chosen the manufacturing of pseudoionone. So far, pseudoionone has been produced industrially in large quantities from an acetoacetic ester with dehydrolinalool at a temperature of 160 °C in Caroll synthesis [29]. Unfortunately, a mixture of diastereomers is formed [30]. Pseudoionone itself is an important precursor of various ionones, which are used to synthesize other products such as vitamin A [31,32,33,34,35,36,37,38]. Instead of producing pseudoionone via the Caroll reaction, an aldol reaction starting with citral and acetone can be employed as depicted in Figure 4. The use of citral, a terpene naturally occurring in several plants, as the carbonyl component of the reaction introduces the renewable resource and safe chemical aspect to the process [39,40]. The alkyl component here is acetone, which is formed as a by-product in equimolar amounts with phenol in the cumene hydroperoxide process. With the increasing demand for phenol, the availability of acetone also increases, which is accompanied by a price reduction [41]. Additionally, bio-based acetone obtained from ABE fermentation can also be used [6].

In order to investigate the behavior of the catalysts in relation to their deactivation effects, they first are tested in a continuous fixed-bed reactor.

## 2. Results and Discussion

### 2.1. Continuous Reaction Operation

**Figure 5:**
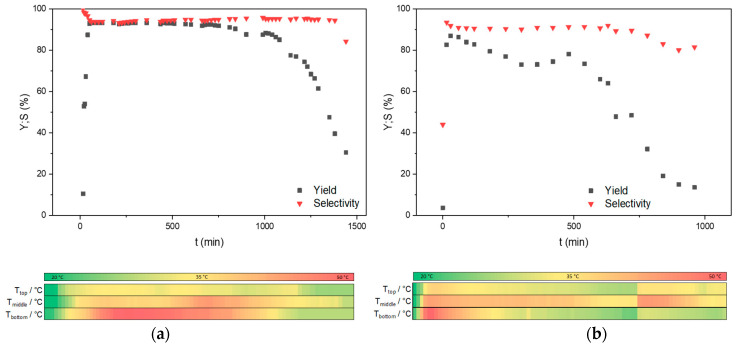
Continuous operation in a fixed-bed reactor with temperature course of (**a**) citral and acetone (1:8) with 2 mL/min and an effective catalyst volume of 11.5 mL Amberlyst^®^ A26 OH catalyst at 30 °C and (**b**) citral and acetone (1:8) with 2 mL/min and an effective catalyst volume of 11.5 mL Lewatit^®^ K 6465 catalyst at 30 °C.

The catalytic performance of Amberlyst^®^ A26 OH and Lewatit^®^ K 6465 was evaluated in a continuously operated fixed-bed reactor, as shown in Section 3.4. The reaction was conducted at a temperature of 30 °C, although the internal temperature locally exceeded this value due to reaction heat, utilizing an effective catalyst volume of 11.5 mL. The experimental results, depicted in Figure 5, illustrate the outcomes. A flow rate of 2 mL∙min^−1^ was maintained for the reaction mixture, composed of citral and acetone in a ratio of 1:8, resulting in a WHSV of 6 h^−1^. The reaction exhibits high yield and selectivity, the latter constantly surpassing 90% over an extended duration. In particular, the Amberlyst^®^ A26 OH catalyst demonstrates prolonged activity, with a decline in both yield and selectivity occurring around the 1100 min mark compared to a decline in the activity of Lewatit^®^ K 6465 occurring after about 500 min.

The obtained results demonstrate contrasting behaviors between Amberlyst^®^ A26 OH and Lewatit^®^ K 6465 regarding the exothermic aldol reaction:

In the case of Amberlyst^®^ A26 OH, the exothermic aldol reaction led to the migration of the hotspot from the lower region (reactor inlet) to the upper region of the reactor, as depicted in the temperature course of Figure 5a. This phenomenon indicates a loss of catalytic activity starting from the bottom, presumably caused by the loss of the catalysts’ active sites.

On the other hand, Lewatit^®^ K 6465 did not demonstrate the migration of a hotspot, as observed in the temperature course of Figure 5b. Consequently, the absence of this phenomenon suggests no catalyst deactivation by the loss of the catalysts’ active sites.

### 2.2. Quantum Chemical Calculations on Catalyst Deactivation

For the Density Functional Theory analysis of the deactivation reactions of both Lewatit^®^ K 6465 and Amberlyst^®^ A26 OH, model structures were built to resemble the area of interest in the ion exchange resin. In these models, the polymer backbone was replaced by a methyl group to produce computationally accessible structures while preserving the functional monomeric unit. Reactions surveyed included S_N_2 deactivations for both Amberlyst^®^ A26 OH and Lewatit^®^ K 6465, as well as the Hofmann Elimination reaction of Lewatit^®^ K 6465. The activation energies of the deactivation reactions are summarized in Table 3. All reactions are predicted to be exothermic and show moderate activation energies. The activation energy for the S_N_2 reaction of the Amberlyst^®^ A26 OH model showed the lowest value with 14.9 kcal/mol, being about ¾ of the value for Lewatit^®^ K 6465 S_N_2 which was 20.9 kcal/mol. The Hofmann elimination showed a slightly lower barrier with 18.8 kcal/mol. This hierarchy does not correspond to the results obtained from continuous experiments in the fixed-bed reactor setup, where Lewatit^®^ K 6465 showed a faster drop in yield. One reason could be the deactivation of Lewatit^®^ K 6465 by other means, e.g., the clogging of pores or changes in the swelling behavior during the catalytic reaction.

**Figure 6:**
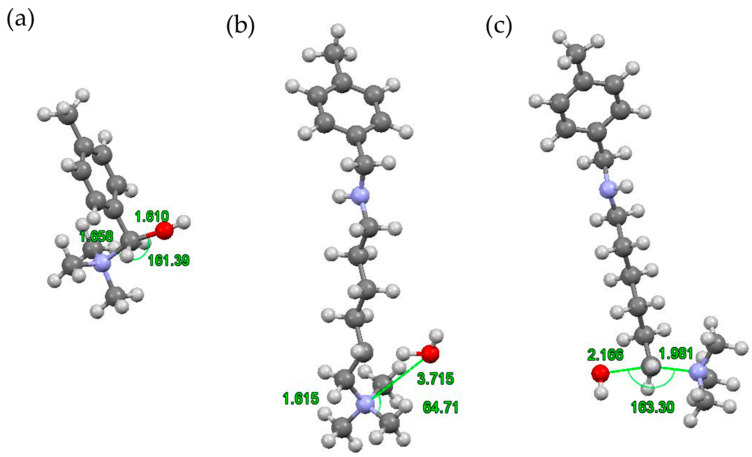
Three-dimensional structures of the transition states of (**a**) model Amberlyst^®^ A26 OH S_N_2, (**b**) model Lewatit^®^ K 6465 Hofmann, and (**c**) model Lewatit^®^ K 6465 S_N_2 deactivations. Atom coloring is H white, C grey, N blue, and O red. Geometries of the transition states are given in the Supplemental Information (Table S3).

The S_N_2 reaction of the Amberlyst^®^ A26 OH model shows the lowest activation energy, possibly due to a stabilizing effect of the phenyl group on the transition state. Figure 6 shows the optimised geometries of the transition states. Of note is the fact that the angle between the participating N, C, and O atoms is approximately 160°, a big step away from the optimal 180°. This can be explained by the steric strain between the methylamine and the phenyl groups. For the Lewatit^®^ K 6465 model, the transition state for the S_N_2 reaction is similarly strained, with a slightly more optimal 163° angle. The methylamine group seems to introduce a steric hindrance to the substitution reaction and might be of interest for the development of more stable ion exchange resins.

The Hofmann elimination reaction of the Lewatit^®^ K 6465 model showed no such strain with the OH- group freely attacking the β-H, as seen in Figure 1b). In this transition state, the H-O-(β-H) angle is similar to that of the resulting water (105° for both). The bond between the terminal carbon and nitrogen of the methyl amine group slightly elongates from 1.52 Å to 1.61 Å before being broken fully.

Overall, the deactivation reactions of both model structures could not confirm the results obtained during the continuous reaction operation. While the faster deactivation of Lewatit^®^ K 6465 could be rooted in the availability of two reaction paths, both exhibit higher activation energies than the one path of deactivation for Amberlyst^®^ A26 OH. The S_N_2 deactivation of Lewatit^®^ K 6465 does show a lower reaction energy, though at the low reaction temperatures observed this cannot overcome the high reaction barrier. Thus, other possibilities must be considered that lead to the observed loss in activity of the Lewatit^®^ K 6465 ion exchange resin.

### 2.3. Batch Experiments

In the subsequent sections, we provide a detailed account of the batch experiments conducted to assess the performance of the catalysts. The reaction was carried out with both catalysts over different time spans, with each point of data representing an individual experiment. For each catalyst a second set of experiments was carried out, using recycled catalyst, each having been used under equal reaction conditions for 240 min.

**Figure 7:**
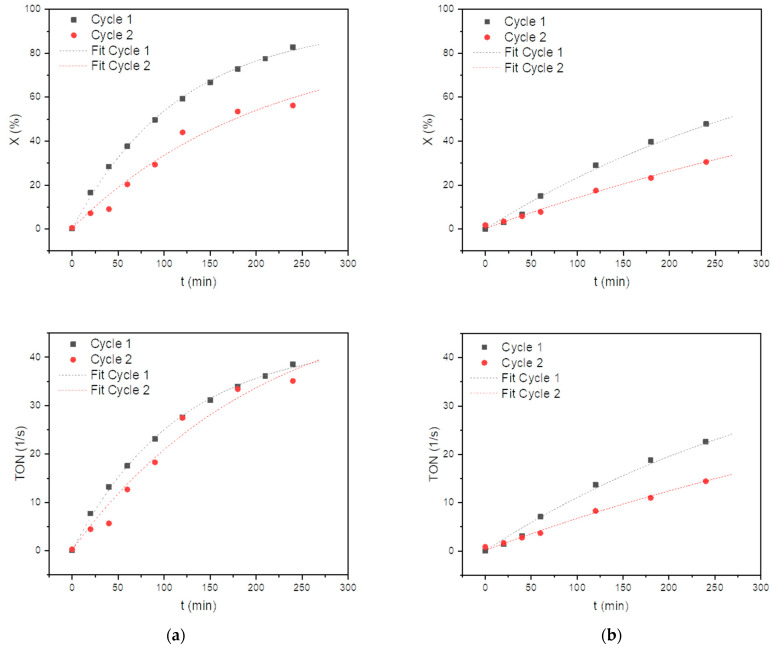
Conversion and Catalytic Performance (TON) over time of (**a**) Amberlyst^®^ A26 OH and (**b**) Lewatit^®^ K 6465 in pseudoionone production.

Amberlyst^®^ A26 OH exhibited a promising catalytic performance throughout the experimental run, as seen in Figure 7a. However, as the reaction progressed, both the conversion and selectivity significantly increased. After 240 min of the first cycle, the conversion of the substrate reached 82.8% and 56.2% after 240 min of the second cycle, while the selectivity towards pseudoionone remained roughly the same with 97.8% after the first cycle and 97.4% after the second. The only by-product observed was diacetone alcohol, which is formed from the aldol reaction of acetone with itself. The observed differences in the catalytic performance between Amberlyst^®^ A26 OH and Lewatit^®^ K 6465 could be attributed to variations in their active site concentrations or structural properties. The active basic capacity of the catalysts, Lewatit^®^ K 6465 and Amberlyst^®^ A26 OH, was determined before and after the reaction, as depicted in Table 4, according to the method described in Section 3.3 [42].

Before the reaction, both Lewatit^®^ K 6465 and Amberlyst^®^ A26 OH exhibited similar levels of activity, with active basic capacities of approximately 0.62 mol/L and 0.63 mol/L, respectively. This proposes that both catalysts possessed a comparable number of active sites capable of participating in the desired transformations. Interestingly, the active basic capacity of Lewatit^®^ K 6465 remained unchanged at 0.62 mol/L after the reaction, indicating its stability and ability to maintain its initial number of catalytic active sites under reaction conditions. This indicates that Lewatit^®^ K 6465 is a robust catalyst with excellent resistance to deactivation by the cleavage of active sites under the reaction conditions employed. In contrast, the active basic capacity of Amberlyst^®^ A26 OH decreased to 0.47 mol/L after the reaction. This reduction in activity advises that the catalyst may have experienced some degree of deactivation or structural changes during the reaction. While both catalysts loose activity during the course of the reaction, only the declined activity of Amberyst^®^ A26 OH can be explained by its deactivation in an S_N_2 reaction of its basic sites. Lewatit^®^ K6465, in contrast, retains its catalytic sites, suggesting deactivation by macroscopic effects such as the blocking of pores by products or side products. Such macroscopic effects are rather associated with properties of the polymer backbone rather than those of its basic sites.

To ascertain whether the pores of the employed catalysts were obstructed, the catalysts were soaked in THF for 24 h following the experiments. The solvent was subsequently analyzed using gas chromatography, revealing that the THF in which Lewatit^®^ K 6465 was immersed exhibited notable amounts of pseudoionone and citral after this period, while the THF containing Amberlyst^®^ A26 OH showed no traces of the utilized reactants. In a further cycle using Lewatit^®^ K 6465 stored in THF, a selectivity of 90% and a conversion of 48% could again be achieved after a duration of four hours. The fact that Amberlyst^®^ A26 OH does not appear to intercalate reactants while Lewatit^®^ K 6465 does is in contrast to the pore volumes and pore diameters reported in Table 1. This may be due to the fact that reactant-induced swelling processes change the pore structures of the catalysts under experimental conditions. A different swelling behavior can be caused by the different water content of both catalysts.

The observed similarity in the initial active basic capacity of both catalysts prior to the reaction underscores their comparable performance potential. However, the contrasting behavior in terms of activity changes after the reaction highlights the distinct characteristics and response of each catalyst under the given reaction conditions.

While Amberlyst^®^ A26 OH loses its active sites due to the exothermic nature of the aldol reaction, it has been demonstrated that the exothermicity of the aldol reaction under our experimental conditions is insufficient to cleave the active sites of Lewatit. In contrast to Amberlyst^®^ A26 OH, the pore structure of Lewatit^®^ K 6465 becomes obstructed under experimental conditions, leading to a significant decrease in its activity. These results indicate the effectiveness of Amberlyst^®^ A26 OH in promoting the desired reaction. The average performance of the active sites is shown as the turnover number, giving back the amount of converted substrate normalized to the number of strong basic sites (TON) [43]. The TON values, which represent the average performance of active sites, steadily increased with time, demonstrating the catalyst’s sustained activity throughout the reaction. Notably, the TON value reached 38.6 at t = 240 min in the first cycle and remained high at 35.1 in the second cycle, indicating the consistently high catalytic efficiency of Amberlyst^®^ A26 OH throughout the experiment, despite a slight decrease between cycles. The nearly identical TON values obtained in both cycles show that all catalytic sites stay accessible.

In contrast, Lewatit^®^ K 6465 exhibited a slightly lower catalytic performance compared to Amberlyst^®^ A26 OH, as shown in Figure 7b. The conversion of the substrate and selectivity towards pseudoionone were lower at each time interval. However, it is important to note that Lewatit^®^ K 6465 still demonstrated considerable catalytic activity, with a conversion of 47.8% and selectivity of 97.0% after 240 min of the first cycle, and a conversion of 30.5% with a selectivity of 91.8% after the second cycle. The TON values for Lewatit^®^ K 6465 ranged from 22.6 after 240 min of the first cycle to 14.4 in the second cycle, indicating a lower activity in the initial experiments with Amberlyst^®^ A26 OH.

## 3. Materials and Methods

### 3.1. Materials

Citral (95%) and Sodium hydroxide was purchased from Acros Organics, Verona, Italy. Acetone (≥99%, laboratory reagen grade) was provided by Fisher Scientific, Waltham, MA, USA. Diacetone alcohol (98%) and mesityl oxide (95%) were provided by TCI Chemicals Europe, Zwijndrecht, Belgium. Pseudoionone (≥90%), tetrahydrofuran (98%), and Sodium hydroxide (reagent grade, ≥98%, pellets (anhydrous)) were obtained from Aldrich Chemistry (Merck, Darmstadt, Germany). Hydrochloric acid (fuming, 37%, for analysis) was obtained from Supelco (Merck, Darmstadt, Germany). Diethyl ether (≥99.8%) was purchased from Honeywell, Charlotte, NC, USA. Hydranal MeOH dry was acquired from Fluka Analytical, Buchs Switzerland and Aqualine™ Complete 5 from Fisher Scientific, Waltham, MA, USA. Amberlyst^®^ A26 OH has been kindly provided by DuPont, Wilmington, DE, USA and Lewatit^®^ K 6465 by LANXESS, Leverkusen, Germany.

### 3.2. Catalyst Activation Procedure (Lewatit^®^ K 6465)

A total of 10 mL of catalyst was placed in a filter tube, covered with glass wool. Then ten times the volume of the catalyst bed of 1 mol/L NaOH was added dropwise at a flow rate of 10 mL/min. The catalyst was then washed with ten times the volume of the catalyst bed of deionized water until the pH at the outlet was neutral. Finally, the catalyst was flushed with five times the volume of its bed with THF. The charging of the Lewatit^®^ K 6465 from the bromide form to the hydroxide form was carried out in accordance with European patent specification EP 1908521 B1 [20].

### 3.3. Characterization of Catalysts

For the determination of ion exchange capacity, about 100 mL of the sample in any chemical state was introduced into a filter tube filled with deionized water without air bubbles and loaded with about 2 mL of 1% hydrochloric acid at a flow rate of 20 mL/min. Thereafter, washing was carried out with deionized water at the same flow rate until a base capacity of ≤0.1 mmol/L up to pH = 4.3 was reached in the washing liquid that had run off. Thus, the resin was in the Cl form. The determination should be started within 24 h. The exchanger, shaken to a constant volume (50 ± 1) mL, was flushed quantitatively with deionized water into the filter tube filled with deionized water and the liquid level was lowered to about 1 cm above the exchanger. Air pockets were to be avoided. Next, 500 mL of 2% strength sodium hydroxide solution was then added to the exchanger with a flow rate of 10 mL/min. Washing was continued with deionized water at the same volume flow rate until the acid capacity in the outflow was ≤0.1 mmol/L up to pH = 8.2. Then, 950 mL sodium chloride solution, 2.5%, was poured through the exchange column and the effluent was collected in a 1000 mL volumetric flask. The volume flow should be around 15 mL/min. It was then washed with deionized water until the mark was filled up and the liquid was mixed well. Finally, 50 mL of this solution was titrated against methyl orange with hydrochloric acid, c(HCl) = 0.1 mol/L [42].

### 3.4. Experimental Setup for Reaction of Acetone and Citral

The discontinuous aldol reaction of citral with acetone was carried out in a 80 mL three-neck flask, connected to a vacuum manifold for inertization with nitrogen, under reflux with 10 mL of catalyst. Then, citral (10 mL) and acetone (80 mL) were added. The reaction was heated to constant 30 or 40 °C and stirred. After 240 min, the reaction was cooled rapidly and a sample was taken.

**Figure 8:**
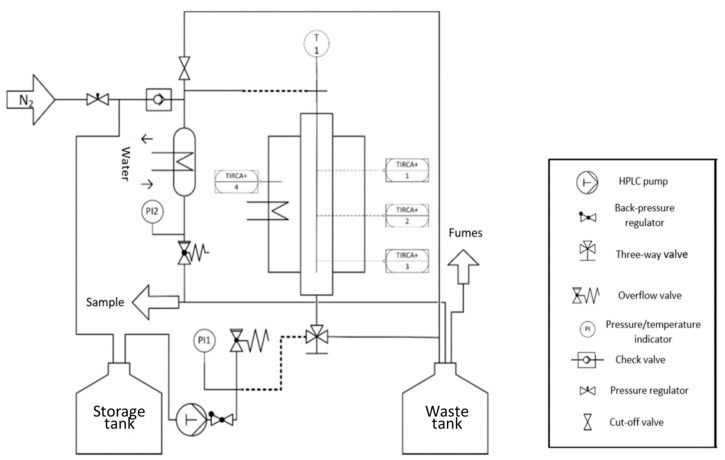
Process flow diagram of the fixed-bed reactor for the aldol reaction of pseudoionone. With PI1: Pressure indication (before reactor); TIRCA+ 1: Temperature indication record control with an alarm if value exceeded at reactor outlet; TIRCA+ 2: Temperature indication record control with an alarm if value exceeded at reactor middle; TIRCA+ 3: Temperature indication record control with an alarm if value exceeded at reactor inlet; TIRCA+ 4: Temperature indication record control with an alarm if value exceeded for reactor heater unit; T1: Temperature indication in the reactor; PI2: Pressure indication (after reactor).

The continuous aldol reaction was carried out in a fixed-bed reactor from HitecZang (Herzogenrath, Germany), as shown in Figure 8. Next, 11.5 mL effective catalyst volume was loaded into the fixed-bed reactor. A precise and controlled feed system using a high-pressure dosing pump was employed to introduce the reactants citral and acetone in a ratio of 1:8 into the fixed-bed reactor. Samples from the aldol reaction were collected at the reactor outlet after a cooling system. Reaction mixtures were analyzed by Gas chromatography using a Shimadzu (Duisburg, Germany) GC-2010-Plus and GCMS-QP2010 SE (to identify and quantify reaction products) equipped with a RTX-200MS column.

### 3.5. Quantum Chemical Calculations on the Catalyst Deactivation

To determine structures and energies of the deactivation reaction steps for Lewatit^®^ K 6465 and Amberlyst^®^ A26 OH, Density Functional Theory (DFT) was employed in the form of the B3LYP functional. The employed basis set was 6-311++g(2d,p) and all calculations were conducted with the Gaussian software (version 09 and 16, Gaussian Inc., Wallingford, CT, USA) [44] on both the RWTH High-Performance Computing cluster as well as the Regional Computing Center (RRZK) at the University of Cologne. As the study was conducted during a system switch from Gaussian 09 to Gaussian 16, both versions were used. Special care was taken to conduct every calculation with the same parameters and no fundamental difference between results of both versions was expected to occur.

Water was used as the solvent via the polarizable continuum model (PCM), as implemented in the Gaussian software. This methodology showed reliable results for similar systems in works by other authors [45].

**Figure 9:**
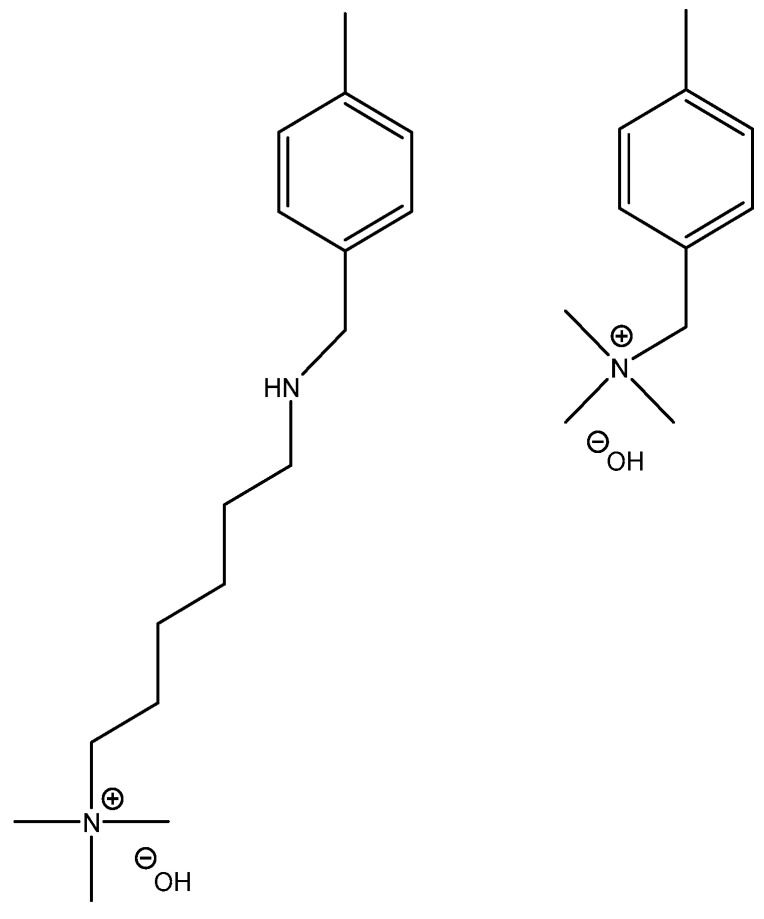
Model geometries for Lewatit^®^ K 6465 and Amberlyst^®^ A26 OH.

Exemplary structures of both Lewatit^®^ K 6465 and Amberlyst^®^ A26 OH were constructed using the monomer containing the catalytically active functional group with the polymer backbone simplified to a methyl group, as seen in Figure 9. The SN2 substitution pathway was investigated for both models and the Hofmann elimination pathway for the Lewatit^®^ K 6465 model. Precursor, transition state, and product molecules were constructed using the Gaussview 6 software (Gaussian Inc., Wallingford, CT, USA). Optimization of the structures occurred via the Berny algorithm, as implemented in the Gaussian program package. Vibrational analysis of the optimized structures showed no negative frequencies for ground state and one vibration with negative frequency for transition states, corresponding to the expected molecular transition.

The change in standard free energy respective to the substrate $∆G_{R}^{0}$ is given for each transformation. The activation energy $E_{A}$ is defined (for reactions with a single transition state) as the difference in standard free energies between substrate ($∆G_{substrate}^{0})$ and transition state ($∆G_{TS}^{0}$):

(1) $$E_{A}=∆G_{TS}^{0}-∆G_{substrate}^{0}$$

This is an important descriptor for the reaction, as it has direct influence on the reaction rate. The overall reaction energy $∆G_{R}^{0}$ is calculated by

(2) $$∆G_{R}^{0}=∆G_{product}^{0}-∆G_{substrate}^{0}$$

Values for the Gibbs free energy are given in the Supplemental Information (Table S2).

## 4. Conclusions

This study has provided groundbreaking evidence that a basic ion exchange resin can retain its active sites under aldol test conditions, thus overcoming a significant challenge in catalysis. The observed deactivation of Lewatit^®^ K 6465 due to pore clogging highlights the need for further optimization in order to mitigate this issue. Interestingly, Amberlyst^®^ A26 OH, despite experiencing a loss of basic sites, has demonstrated the retention of accessibility for these sites even after repeated use. This remarkable finding suggests the potential for designing a hybrid catalyst incorporating the robust backbone of Amberlyst^®^ A26 OH and the active sites of Lewatit^®^ K 6465, which is expected to exhibit exceptional longevity and hold immense promise for industrial applications. The calculated activation energies for the deactivation reactions of the Hofmann elimination and S_N_2 reaction have shed light on the potential advantages of Lewatit^®^ K 6465 in terms of lifespan, underlining its stability in maintaining activity. Notably, both catalysts initially exhibited a similar number of active sites before the reaction, indicating that the observed differences in longevity are not solely attributed to the initial population of active sites. The normalization of substrate conversion to the number of active sites clearly suggests that the decline in the conversion rate of Amberlyst^®^ A26 OH corresponds directly to the loss of its active sites. In contrast, the unaltered number of basic sites of Lewatit^®^ K 6465 throughout the reaction implies that Lewatit has the potential to be a long-term stable catalyst for aldol reactions. In order to achieve this objective, it is imperative to address the challenge of catalyst pore blockage.

## Figures and Tables

**Table 1:** Comparison of physical and chemical properties: Amberlyst**^®^** A26 vs. Lewatit**^®^** K 6465 [21,22].

	Amberlyst^®^ A26 OH	Lewatit^®^ K 6465
**Physical Properties** Copolymer Matrix Functional Group Physical Form	Styrene-divinylbenzene Macroporous Quaternary ammonium, Type I Tan, opaque, spherical beads	Styrene-divinylbenzene Macroporous Quaternary ammonium, Type I Beige, opaque, spherical beads
**Chemical Properties** Ionic Form as Shipped Concentration of Base Sites Water Retention Capacity	OH^−^ ≥0.80 eq∙L^−1^ 66–75%	Br^−^ ≥1.6 eq∙L^−1^ 52–58%
**Particle Size** Particle Diameter Uniformity Coefficient	560–700 μm ≤1.45	700 µm (±50) max. 1.1
**Density** Shipping Weight	675 g∙L^−1^	800 g∙L^−1^
**Surface data** BET surface area Total pore volume Average pore diameter	30 m²/g 0.2 mL/g 290 Å	40 m²/g 0.6 mL/g 630 Å

**Table 2:** Active site structure of Lewatit^®^ K 6465 and Amberlyst^®^ A26 OH; red = crosslinker; blue = active site.

Backbone	Catalyst	R_1_
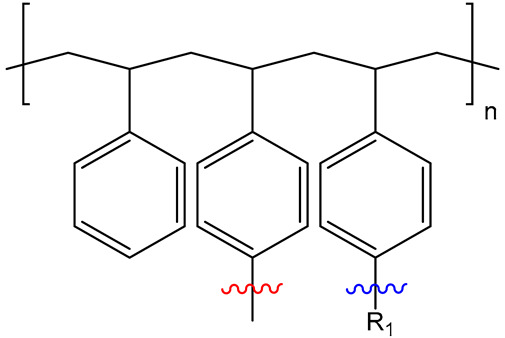	Lewatit^®^ K 6465	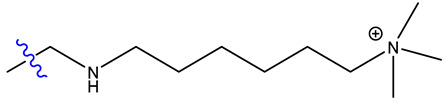
	Amberlyst^®^ A26 OH	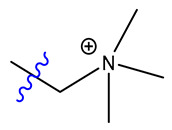

**Table 3:** Activation energies as determined by DFT calculations on model structures.

Model	Reaction	E_A_ [kcal/mol]
Amberlyst^®^ A26 OH	S_N_2	14.9
Lewatit^®^ K 6465	S_N_2	20.9
	Hofmann	18.8

**Table 4:** Characterization of active basic sites before and after the reaction via titration using 100 mL of Lewatit^®^ K 6465 and Amberlyst^®^ A26 OH and 1% hydrochloric acid at a flow rate of 20 mL/min.

Catalyst	Active Basic Capacity (Before Reaction) [mol/L]	Active Basic Capacity (After Reaction) [mol/L]	Loss [%]
Lewatit^®^ K 6465	0.62	0.62	0
Amberlyst^®^ A26 OH	0.63	0.47	25.4

## Data Availability

Data is contained within the article or Supplementary Material.

## Supplementary Materials

The following supporting information can be downloaded at: https://www.mdpi.com/article/10.3390/ijms241814367/s1.
