# Supplementary material for: Temperature Stable Ion Exchange Resins as Catalysts for the Manufacturing of Vitamin Precursors by Aldol Reaction
Source: Int J Mol Sci. 2023 Sep 21;24(18):14367. doi: 10.3390/ijms241814367 (PMC10532307; doi:10.3390/ijms241814367)
Supplement: Supplementary file 1 [file ijms-24-14367-s001.zip › ijms-2613604-SI.pdf]

International Journal of
Molecular Sciences

MDPI

Supplementary materials

The following mathematical fits for the experiments of Amberlyst® A26 OH and Lewatit® K6465 where calculated according to the eq. S.1., as seen below.

$$X_t = X_{max} \cdot \left(1 - e^{(-k \cdot t)}\right) \qquad \text{eq. S.1.}$$

The results of the fit analysis for both catalysts in a first and second reaction cycle are depicted in Figure S1 and Table S1 and Table S2.

a)

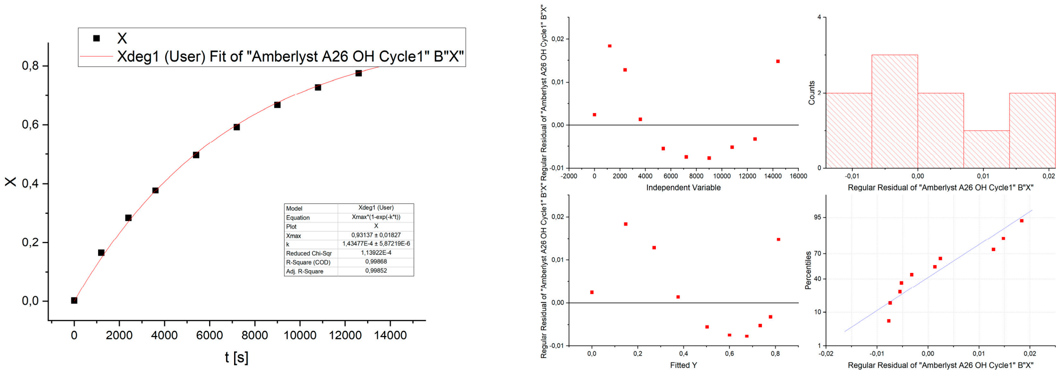

b)

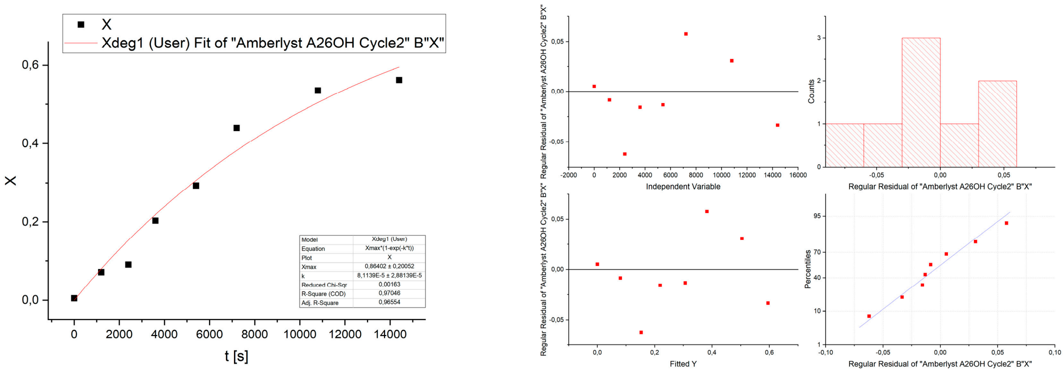

c)

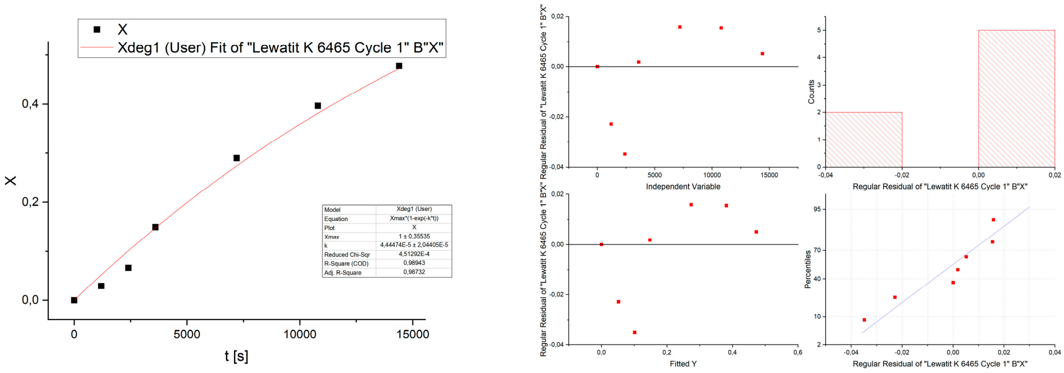

d)

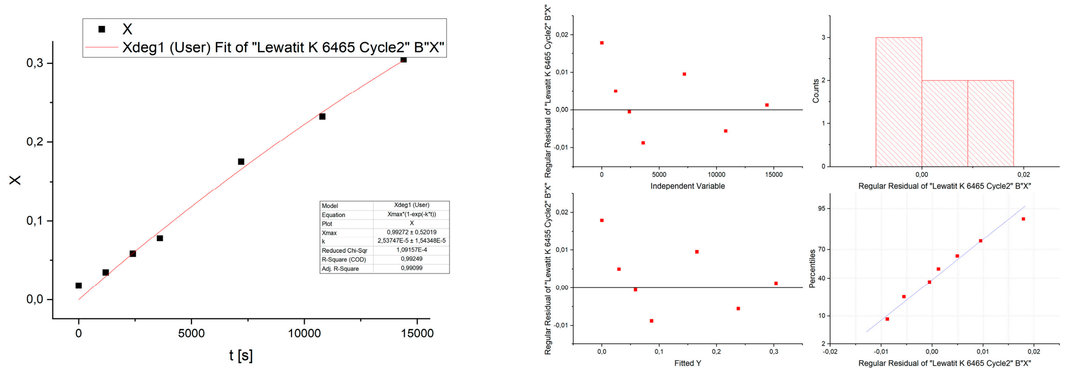

Figure S1. Fitted Reaction Progress Analysis for Amberlyst® A26 OH in a) first and b) second cycle and Lewatit® K6465 in c) first and d) second cycle.

Table S1. Comparative Analysis of Catalytic Performance for Amberlyst® A26 OH and Lewatit® K6465: X_{max}, Rate Constants, R-squared, and Chi-squared Values.

Catalyst	$X_{max.}$	k / s^{-1}	R^2	χ^2
Amberlyst® A26 OH Cycle 1	0.93137	1.43 E-04	0.99868	1.13922 E-04
Amberlyst® A26 OH Cycle 2	0.86402	8.11 E-05	0.97046	0.00163
Lewatit® K6465 Cycle 1	1	4.44 E-05	0.98943	4.51292 E-04
Lewatit® K6465 Cycle 2	0.99272	2.54 E-05	0.99249	1.09157 E-04

Table S2. Activation energies and reaction enthalpies as determined by DFT calculations on model structures.

Model	Reaction	E_A [kcal/mol]	ΔG_R^0 [kcal/mol]
Amberlyst® A26 OH	S_N2	14.9	-36.0
Lewatit® K 6465	S_N2	20.4	-40.7
	Hofmann	22.7	-34.8

Table S3. Geometries of transition states.

a) Model Amberlyst® A26 OH S_N2

C	-3.07041	-0.04693	0.00121
C	-4.56586	-0.23015	-0.00291
C	-2.35474	0.02776	1.19816
H	-5.03051	0.32026	-0.82297
H	-5.01016	0.1071	0.93452
H	-4.82697	-1.28524	-0.13073
C	-2.35494	0.04833	-1.19565

C	-0.97312	0.17987	1.20086
H	-2.88558	-0.02044	2.14231
C	-0.97439	0.19974	-1.19626
H	-2.88632	0.01675	-2.14037
C	-0.25781	0.25462	0.00304
H	-0.45164	0.25876	2.14796
H	-0.45279	0.29408	-2.14187
C	1.228	0.47715	0.00299
H	1.61411	0.57917	-1.00764
H	1.61785	0.56286	1.0137
N	1.77692	-1.08723	-0.00385
C	1.30256	-1.82136	-1.22298
C	1.3266	-1.82308	1.22345
C	3.27893	-1.03476	-0.01875
H	1.6419	-1.28904	-2.10785
H	1.72301	-2.82388	-1.20736
H	0.21825	-1.87317	-1.20676
H	1.68492	-1.29327	2.10239
H	1.7447	-2.82642	1.19721
H	0.2421	-1.87295	1.22919
H	3.60207	-0.51053	-0.91417
H	3.66444	-2.05121	-0.01932
H	3.61966	-0.50442	0.86648
O	1.2075	2.08711	0.00698
H	1.08953	3.04527	0.00826

b) Model Lewatit® K 6465 Hofmann-elimination

N	2.73302	-0.79042	0.42676
C	1.63834	0.0225	-0.09503
C	0.29671	-0.67493	0.10899
C	-0.88615	0.14268	-0.41507
C	-2.23707	-0.56223	-0.26161
C	-3.41378	0.26029	-0.80075
C	-4.6466	-0.52986	-1.01578
N	-5.85944	-0.28142	0.02188
C	-6.93302	-1.2757	-0.26389
C	-5.36801	-0.45476	1.41886
C	-6.39788	1.0986	-0.15913
H	2.5714	-1.78373	0.3287
H	1.63015	0.98419	0.42714
H	1.78243	0.24239	-1.16348
H	0.16002	-0.88169	1.17611

H	0.31933	-1.64724	-0.39825
H	-0.72417	0.37309	-1.47541
H	-0.91734	1.10685	0.10475
H	-2.3793	-0.79513	0.8036
H	-2.15824	-1.54655	-0.76075
H	-3.13501	0.72585	-1.75258
H	-3.47885	1.58354	0.15663
H	-5.1424	-0.32652	-1.96632
H	-4.52029	-1.61567	-0.91552
H	-7.77221	-1.10656	0.40828
H	-7.25783	-1.15823	-1.29569
H	-6.53772	-2.27811	-0.11489
H	-6.19033	-0.2793	2.11093
H	-4.56799	0.26	1.58846
H	-4.9972	-1.47107	1.53524
H	-6.78235	1.19123	-1.17296
H	-7.20243	1.2619	0.55627
H	-5.59319	1.8093	0.00486
O	-3.48705	2.46463	0.81522
H	-2.63961	2.90074	0.66627
C	4.13646	-0.4101	0.21091
H	4.35835	-0.4441	-0.83528
H	4.29756	0.58211	0.57761
C	5.05552	-1.39076	0.96273
C	5.55895	-2.51566	0.30879
C	5.38475	-1.15435	2.29737
C	6.39087	-3.40426	0.98962
H	5.29858	-2.70216	-0.74319
C	6.21771	-2.0426	2.97826
H	4.98821	-0.26771	2.81287
C	6.72067	-3.1675	2.32468
H	6.7872	-4.29127	0.47443
H	6.47745	-1.85573	4.03044
C	7.63934	-4.14882	3.07612
H	7.50238	-5.13564	2.68581
H	8.65936	-3.85256	2.94694
H	7.39457	-4.1401	4.11771

c) Model Lewatit® K 6465 S_N2

C	1.18381	1.0171	-0.00139
H	1.63123	1.33431	-0.92445
H	1.63469	1.34086	0.91752

C	-5.23354	0.81939	0.35856
H	-5.08386	0.73428	1.41974
H	-6.1476	1.26517	0.01204
N	-6.08388	-0.96877	0.30624
C	-5.1269	-2.03954	0.63857
C	-6.68783	-1.19175	-1.01978
C	-7.1347	-0.88143	1.33771
H	-4.66859	-1.82935	1.60404
H	-5.63647	-3.00676	0.69023
H	-4.3529	-2.09194	-0.12466
H	-7.36543	-0.37111	-1.25314
H	-7.24876	-2.13087	-1.03336
H	-5.9093	-1.23964	-1.77893
H	-6.67701	-0.69978	2.30929
H	-7.70829	-1.81128	1.38327
H	-7.80927	-0.05932	1.10139
O	-4.79708	2.89768	0.78608
H	-5.49354	3.10102	1.42339
C	-4.05814	0.72083	-0.57667
C	-2.70133	0.57848	0.11892
H	-4.19663	-0.1122	-1.2728
H	-4.0602	1.62303	-1.19033
C	-1.53664	0.48555	-0.86951
H	-2.56025	1.43816	0.77738
H	-2.70086	-0.31136	0.75775
C	-0.17219	0.38848	-0.18287
H	-1.67991	-0.3846	-1.52136
H	-1.5487	1.36432	-1.52528
C	0.98717	0.2771	-1.16837
H	-0.01628	1.26711	0.45179
H	-0.16214	-0.48318	0.48301
N	2.27947	0.2216	-0.47856
H	0.99759	1.15334	-1.82569
H	0.83299	-0.60045	-1.81898
H	2.27123	-0.5599	0.17253
C	3.40948	0.04426	-1.40183
H	3.28089	-0.84303	-2.0415
H	3.42755	0.91283	-2.06735
C	4.72117	-0.0599	-0.66199
C	5.34825	1.07902	-0.14708
C	5.3319	-1.29428	-0.45009
C	6.54288	0.98247	0.55409

H	4.89418	2.05228	-0.29995
C	6.53049	-1.39143	0.2559
H	4.86913	-2.19408	-0.84215
C	7.15766	-0.25677	0.76926
H	7.00942	1.88323	0.93947
H	6.98278	-2.36589	0.40618
C	8.4632	-0.35063	1.51875
H	8.72842	-1.38835	1.72522
H	9.28059	0.09469	0.94393
H	8.41311	0.18328	2.47095
